# Assessment of staffing needs for registered nurses and licensed practical nurses at primary care units in Brazil using Workload Indicators of Staffing Need (WISN) method

**DOI:** 10.1186/s12960-021-00674-0

**Published:** 2022-01-28

**Authors:** Daiana Bonfim, Ana Carolina Cintra Nunes Mafra, Danielle da Costa Palacio, Talita Rewa

**Affiliations:** Hospital Israelita Albert Einstein. Center for Studies, Research and Practice in PHC and Health Care Network (CEPPAR), Av. Albert Einstein 627, São Paulo, São Paulo 05652-900 Brazil

**Keywords:** Primary health care, Human Resources in Health, Nursing, Health services

## Abstract

**Background:**

The balance between supply and demand for primary health care (PHC) services is one of the main challenges to the health system in Brazil. In this context, the application of planning methods could benefit the decision-making process for human resources organizations. Hence, the objective of this study was to assess the staffing needs for registered nurses (RNs) and licensed practical nurses (LPNs) at PHC services using the WISN method.

**Methods:**

The Workload Indicators of Staffing Need (WISN) methodology was applied at 13 Primary Care Units (PCU) located in the city of São Paulo, Brazil. It included 87 RNs and 174 LPNs, and used data from 2017 to 2019.

**Results:**

The WISN results found that RNs were under high workload pressure at 10 PCUs (77%) in 2017 and 2018, with a decrease to 8 PCUs (61%) in 2019. For LPNs, high workload pressure increased from 2 PCUs (15%) in 2017 to 13 PCUs (100%) in 2018, with a decrease to 11 (85%) in 2019.

**Conclusion:**

The assessment of staffing needs for RNs and LPNs at the PCUs included in the study identified a consistent deficit in the number of professionals, and high workload pressure in most services throughout the study period.

## Background

Balancing the supply of healthcare professionals against the growing demand of patients is one of the biggest challenges for primary health care (PHC) systems around the world. In this context, the use of workforce planning methods could contribute to the analyses and decision-making process to allocate the right number of professionals in the right place, at the right time, in order to meet the health needs of specific populations.

Health workforce planning is understood as a strategic function and a continuous and iterative process, with investigations and analyses of the quantity and quality of workers, supported by data that reflect both planned and unplanned changes at the various determining levels of supply and demand [1, 2].

Some human resource planning methods, such as the Workload Indicators of Staffing Need (WISN) [3], have been used in different regions of the world, in PHC, hospital, and outpatient services for decision-making and planning at local, regional, and national levels [4]. Studies conducted in urgency and emergency services demonstrated that the WISN tool is simple and easy to use, can measure direct and indirect nursing activities and translate the workload. In addition, it can be used to expand the role of the nursing staff and define strategies to increase the efficiency of units [5].

In Brazil, PHC managers make their staffing decisions based on two criteria: following guiding policies, such as the National Policy for Primary Care (PNAB, acronym in Portuguese) [6], and on professional experience and/or judgment. The end result is a fragile planning for staffing needs, and little optimization of financial resources.

The PNAB establishes the composition of PHC teams, which are composed of a registered nurse (RN), a physician, two licensed practical nurses (LPNs), and a varied number of community health workers (CHW), that are organized geographically covering populations of 2000 to 3500 individuals each, with no overlap or gap between coverage areas. Oral health teams, composed of a dental surgeon and an oral health technician, endemic diseases control agents, and a manager can also be added to PCUs to work alongside PHC teams [6].

The PNAB criteria for staffing PCUs is mainly based on the size of the population covered, and do not consider varying workloads produced by different population profiles, nor different regional and local characteristics that should guide the structuring of services and the organization of work processes [7].

The Brazilian Federal Council of Nursing, through Resolution no. 543 of 2017, [8] has established staffing parameters for different nursing categories and healthcare settings based on the application of the adapted WISN method, as well as the definition of direct care for health service activities, and indirect care for support activities and additional activities [8].

In spite of published resolutions and policies, PHC continues to suffer from a growing gap between the demand for services and the number of professionals available [9]. This situation is fueling a growing debate on the need to expand the scope of practice of PHC nurses to increase universal access to health and improve quality of care [10]. To reach these goals it would be important to combine the efforts of changing the scope of nursing practice and of implementing evidence-based nursing staffing methodologies for PHC.

Hence, the objective of this study was to assess the staffing needs for RNs and LPNs at PHC services using the WISN method.

## Methods

### Scope and setting

The WISN methodology was applied to two large districts of the city of São Paulo, Brazil, covered by 87 PHC teams distributed across 13 PCUs serving approximately 270 000 individuals. Management of these services is done through a public–private partnership between the Municipal Health Department and the *Instituto Israelita de Responsabilidade Social Albert Einstein*.

The study included data from 87 RNs and 174 LPNs that were working at the 13 PCUs included in the study between 2017 and 2019.

### WISN calculation

The available working time (AWT) was obtained through institutional data provided by the human resources system. The absences on working days related to vacation, sick leave and other leave (including provided trainings) were considered, as well as national and local holidays and long weekends. All nursing professionals were hired to work 8 h per day, 5 days per week.

The time in minutes was converted into hours, and the workload components were defined as proposed by Bonfim et al*.* [11] This study defined parameters for average times and activities using a work sampling technique, and included 32 613 non-participatory observations of activities performed by nurses working as part of PHC teams in five Brazilian regions. As a result, it made it possible to estimate time patterns for activities that are usually registered in administrative databases, as well as for activities that are not, facilitating the use of human resource planning equations in Brazil [11].

Health Service Activities were considered to be direct care activities, that is, those performed by all nursing professionals in the presence of patients, and that were recorded on a productivity sheet. Support activities and additional activities were considered to be indirect care activities, that is, activities that would benefit patients, but would not require their presence, and usually were not recorded on a productivity sheet. Productivity data were extracted from an administrative PHC database. Vaccination activities were assigned to LPNs, since these tasks are performed by them most of the time, and it was not possible to link individual activity to an individual professional.

For educational groups activities, the number of meetings conducted and not the number of patients attending, was considered. For appointments, home visits, outpatient procedures, monitoring of vital signs, administration of medications, venipuncture, and unscheduled care, the number of services delivered was considered.

For support activities and additional activities, the percentage of work time defined by Bonfim et al*.* [11] was used.

### Data analysis

The available working time (AWT) was calculated according to the WISN manual [3]. To calculate the necessary number of professionals to conduct needed health services, activities were considered to be direct care activities ($Q_{direct \,care}$), and the following equations were used:

$$Q_{direct \,care} = q_{1} + q_{2} + \ldots + q_{n} ,$$

$$where\,q_{i} = \frac{{P _{i} \times T_{i} }}{AWT},$$

*i* (*i* = 1, 2, …, *n*) = workload component, $P _{i}$ = annual workload, $T_{i}$ = standard workload (hours), the calculation of indirect care ($Q_{indirect\, care } \%$), which included support activities and additional activities components, used the percentage of participation of nursing professionals, obtained by adding up the percentages of participation of each category, as proposed by Bonfim et al. [11]

The quantity *Q* for the nursing category being evaluated was calculated by the equation:

$$Q = \frac{{Q_{direct\,care} }}{{1 - \frac{{Q_{indirect\, care } \% }}{100}}}.$$

The difference between current and required staffing levels can identify under- or overstaffing, while the WISN ratio can evaluate the level of daily work pressure among employees. A WISN ratio greater than 1 indicates a surplus of employees, and a ratio smaller than 1 indicates a shortage of employees. Whenever the ratio is smaller than 1, it indicates that working pressure is high [3].

### Ethics approval

The research project was approved by the Research Ethics Committee (protocol CAAE: 23388819.9.0000.0071) according to Resolution 466/12 of the Brazilian National Health Council.

## Results

The found AWTs ranged from 1402.48, for RNs in PCU M, to 1804.48, for LPNs in PCU H. There was an increase in other leaves of absence in 2019 because of trainings offered by the organization (Table 1).

**Table 1:** Available working time (AWT) of registered nurses and licensed practical nurses in 2017, 2018 and 2019

Unit	Professional category	Absent days due to holidays in the year			Working days in vacations			Days on leave		
		2017	2018	2019	2017	2018	2019	2017	2018	2019
PCU A	RN	17	13.5	15.5	14	25	17	6.6	8	2.6
	LPN	17	13.5	15.5	15.2	23.7	13.1	7.4	14.4	6.3
PCU B	RN	17	13.5	15.5	10.6	17.6	16.2	2	5.2	3
	LPN	17	13.5	15.5	18.7	33	18.8	2.4	3.6	6.3
PCU C	RN	17	13.5	15.5	13.1	23.8	9.4	3.9	5.8	1
	LPN	17	13.5	15.5	18	42.6	13.4	4.4	2.3	5.1
PCU D	RN	17	13.5	15.5	15.3	24.2	16.3	1	0	2.8
	LPN	17	13.5	15.5	15.3	31.3	15.8	2.1	4.2	0.9
PCU E	RN	17	13.5	15.5	20.9	36.7	28.6	3.6	1.5	0.6
	LPN	17	13.5	15.5	19.9	30.2	24	2.9	5.4	1.4
PCU F	RN	17	13.5	15.5	12.8	29.1	12.5	4.2	4.5	2.6
	LPN	17	13.5	15.5	16.8	29.9	13.2	3.9	5.5	4.4
PCU G	RN	17	13.5	15.5	11.2	29.2	14.4	7.4	0.4	6.6
	LPN	17	13.5	15.5	23.3	32.3	22	2.5	4.8	17
PCU H	RN	17	13.5	15.5	20.8	22	18.5	6.2	7.3	12.3
	LPN	17	13.5	15.5	18.5	31.1	12.3	3	4	1.1
PCU I	RN	17	13.5	15.5	11.8	28.1	19.2	2.2	5.5	3.7
	LPN	17	13.5	15.5	17.1	26.5	10.5	2.4	4.2	9.3
PCU J	RN	17	13.5	15.5	17.4	21.5	15.7	4	0.6	1.3
	LPN	17	13.5	15.5	14.6	22.9	14.9	4.9	3.8	5.6
PCU L	RN	17	13.5	15.5	16.8	34	15.3	4.5	5	4.2
	LPN	17	13.5	15.5	15.4	28.3	12.3	3.7	3.1	4.4
PCU M	RN	17	13.5	15.5	16.7	30.2	23.4	2.7	2.5	40.4
	LPN	17	13.5	15.5	15.3	22.9	35.4	2.9	2.2	3.6
PCU N	RN	17	13.5	15.5	10.6	18.5	14.1	3.6	0	0
	LPN	17	13.5	15.5	20.3	26.9	11.4	1.1	5.6	5.7

Unit	Professional category	Absent days due to other leaves in the year			Working hours/day			AWT		
		2017	2018	2019	2017	2018	2019	2017	2018	2019
PCU A	RN	6	4.5	9.2	8	8	8	1732	1672	1726
	LPN	3.6	2.7	8.2	8	8	8	1734	1646	1735
PCU B	RN	4.4	8.2	22	8	8	8	1808	1724	1626
	LPN	5.1	7.1	10.1	8	8	8	1735	1622	1675
PCU C	RN	4.8	6.3	15.8	8	8	8	1770	1685	1746
	LPN	5.3	5.7	7.1	8	8	8	1723	1567	1752
PCU D	RN	4.5	8.2	17	8	8	8	1778	1713	1668
	LPN	6.1	8.3	7.4	8	8	8	1756	1622	1764
PCU E	RN	8.1	6	21.3	8	8	8	1684	1618	1553
	LPN	6.6	6.7	28	8	8	8	1709	1634	1528
PCU F	RN	5.9	5.7	17.4	8	8	8	1761	1658	1696
	LPN	4.8	4.6	8.8	8	8	8	1740	1652	1745
PCU G	RN	5.6	6.2	14	8	8	8	1751	1686	1676
	LPN	4.7	7.4	10.5	8	8	8	1700	1616	1560
PCU H	RN	5.1	6.3	9.1	8	8	8	1687	1687	1637
	LPN	5.9	4.8	5.6	8	8	8	1725	1653	1804
PCU I	RN	6.9	5.2	22.8	8	8	8	1777	1662	1591
	LPN	5.1	5.1	11.7	8	8	8	1747	1686	1705
PCU J	RN	3	14.2	12.8	8	8	8	1749	1682	1718
	LPN	3.5	6.4	15.8	8	8	8	1760	1707	1665
PCU L	RN	7.9	7.4	19.5	8	8	8	1710	1601	1644
	LPN	5.6	7.8	9	8	8	8	1747	1658	1751
PCU M	RN	5.4	2.9	5.4	8	8	8	1745	1687	1402
	LPN	3.6	5.5	9.6	8	8	8	1769	1727	1567
PCU N	RN	7	13.7	10.2	8	8	8	1775	1714	1761
	LPN	4.2	12.9	17.5	8	8	8	1739	1609	1679

RN, registered nurses; LPN, licensed practical nurses. Number of weeks in the year = 52. Number of working days in the week = 5

The activity with the greatest workload volume for RNs was appointments, with 207 008 in 2017; 205 365 in 2018; and 184 725 in 2019. For LPNs, there was an important increase in the vaccination activity jumping from 80 143 in 2017 to 256 194 in 2018, and 224 436 in 2019 (Table 2).

**Table 2:** Annual workload of registered nurses and licensed practical nurses in 2017, 2018 and 2019

Unit	Professional category	Consultation			Outpatient procedures			Home visit			Support to exams			Promotion of education actions		
		2017	2018	2019	2017	2018	2019	2017	2018	2019	2017	2018	2019	2017	2018	2019
PCU A	RN	10864	11250	10368	–	–	2	943	874	864	1	–	–	324	169	147
	LPN	–	–	–	1229	850	1185	7498	5643	5915	180	321	375	196	33	–
PCU B	RN	14235	11352	11617	–	16	31	639	526	336	–	371	807	1323	771	189
	LPN	–	–	–	591	1049	1159	7911	7183	5522	689	1201	1023	89	8	1
PCU C	RN	18337	18886	16889	–	4	72	2425	2275	2301	43	117	150	200	28	375
	LPN	–	–	–	2601	3279	3102	11974	11598	11710	963	2365	1399	–	1	–
PCU D	RN	7508	11227	9006	–	–	–	482	366	141	6	23	496	81	6	–
	LPN	–	–	–	1031	914	1719	2976	2678	3310	650	579	370	9	3	–
PCU E	RN	17781	17677	19498	–	–	7	2728	2401	2036	280	433	1268	217	127	151
	LPN	–	–	–	1285	1539	1588	11023	9294	4663	1538	1390	630	254	125	78
PCU F	RN	27814	23323	21585	–	1	19	2522	2176	1744	346	559	125	333	227	180
	LPN	–	–	–	3532	2416	1901	15431	13744	9215	756	1245	561	15	–	–
PCU G	RN	17398	19687	14050	–	16	4	859	942	761	–	186	888	13	25	36
	LPN	-	-	-	1316	1752	2169	7304	7270	5165	928	1813	1515	57	52	7
PCU H	RN	14902	14245	10718	176	59	70	1880	1040	1370	125	562	1933	112	115	190
	LPN	–	–	–	2265	2007	2434	8006	6012	6329	834	1487	471	–	–	–
PCU I	RN	19268	16013	13144	1	–	16	1371	986	1694	319	372	1489	64	23	3
	LPN	–	–	–	1171	1321	1512	8718	5766	2858	195	419	355	–	–	–
PCU J	RN	11493	12630	13068	–	–	3	217	456	1558	2	303	1336	44	53	280
	LPN	–	–	–	481	621	1094	7206	8106	9250	571	339	725	59	26	–
PCU L	RN	19195	20869	17284	–	–	1	1723	1189	2692	–	126	32	38	175	6
	LPN	–	–	–	2027	1996	2175	15454	13613	10572	793	665	757	1	–	–
PCU M	RN	13862	14629	14276	18	12	26	2354	2304	2240	134	197	1545	167	61	58
	LPN	–	–	–	2028	1178	1699	11115	11205	11085	209	198	427	133	75	1
PCU N	RN	14351	13577	13222	–	3	57	1120	1262	1296	157	303	701	230	8	31
	LPN				620	726	661	7795	7647	7329	332	609	692	171	60	13

Unit	Professional category	Administration of medications			Control of immunization and vaccination			Vital signs, weight and height measurements			Venopuncture: venous blood sample			Care delivered to spontaneous demand		
		2017	2018	2019	2017	2018	2019	2017	2018	2019	2017	2018	2019	2017	2018	2019
PCU A	RN	–	–	–	–	–	–	–	–	–	–	–	–	–	–	–
	LPN	3203	2774	2930	3377	12265	12686	4325	2973	3605	7526	6474	6855	–	–	–
PCU B	RN	–	–	–	–	–	–	–	–	–	–	1	8	3	486	8
	LPN	1852	2815	2916	4224	13613	15056	11935	7676	6412	4638	4014	3592	–	–	–
PCU C	RN	–	–	3	–	–	–	–	–	1	60	–	–	105	328	34
	LPN	4924	7436	6552	4642	26453	19266	6047	6364	4455	9992	8473	7204	–	–	–
PCU D	RN	–	–	–	–	–	–	–	–	–	–	–	–	5485	21	9
	LPN	6086	13934	14656	4232	10209	12258	6498	5914	4089	5101	4117	4208	–	–	–
PCU E	RN	1	1	–	–	–	–	–	–	–	–	–	1	8	16	7
	LPN	12119	13554	15597	6806	18212	14339	8289	5517	5278	6490	5557	5668	–	–	–
PCU F	RN	–	–	–	–	–	–	–	24	2	–	–	1	276	6	–
	LPN	10854	8123	7578	6257	25959	20668	11631	8987	5558	10347	9226	9386	–	–	–
PCU G	RN	–	–	–	–	–	–	–	–	–	–	–	–	–	–	101
	LPN	4377	5246	6067	4627	16058	15154	6496	5598	6390	4981	5949	6667	–	–	–
PCU H	RN	–	–	–	–	–	–	–	–	–	469	45	4	635	387	175
	LPN	2532	3734	4030	4867	16104	15580	5378	4491	3326	7277	7946	4836	–	–	–
PCU I	RN	–	–	–	–	–	–	–	–	–	–	–	–	138	100	30
	LPN	3335	3782	4466	5333	17515	16133	4273	3657	3227	4672	5394	5069	–	–	–
PCU J	RN	–	–	–	–	–	–	–	–	–	–	–	–	2829	1433	75
	LPN	2022	3896	4434	4629	16098	16920	5050	3853	5006	3230	4923	5991	–	–	–
PCU L	RN		12	–	–	–	–	39	5	1	5	–	–	–	–	–
	LPN	5254	4468	5539	5850	30192	29919	12417	8368	5882	9536	7260	8109	–	–	–
PCU M	RN	–	–	–	–	–	–	–	–	1	–	–	–	38	–	1
	LPN	3431	1009	3028	6570	16183	11950	10684	6758	6588	7093	5202	5443	–	–	3
PCU N	RN	–	–	–	–	–	–	–	–	238	–	–	–	–	–	103
	LPN	2279	2775	3395	18729	37333	24507	2961	4077	3919	5575	4337	4901	–	–	–

RN, registered nurses; LPN, licensed practical nurses

The percentage of support and additional activities for RNs and LPNs was 37.4% and 24.7%, respectively (Table 3).

**Table 3:** Support activities and Additional activities of registered nurses and licensed practical nurses in 2017, 2018 and 2019, proposed by Bonfim et al*.* [11]

Activities [11]	Registered nurse (%)	Licensed ractical nurse (%)
Education actions for healthcare workers	2.1	1.4
Infection control	0.1	1.4
Control of supplies	0.5	3.6
Organization of working process	3.7	1
Documentation	12.4	9.3
Mapping and territorialization	0.1	0
Referral and Contra-referral	0.3	0.3
Administrative meeting	5.9	1.4
Meeting to assess professional care	1.9	1
Supervision of works at the unit	0.4	0
Sharing information on health care	6.2	3
Interpretation of laboratory data	0.2	0
Health surveillance	1.3	0.4
Support to students	1	0.3
Development of administrative process/routine	0.3	0.1
Orientations on health system	1	1.5
Sums	37.4	24.7

The WISN calculations found that in 2017 RNs were under a high workload pressure at 10 PCUs (77%), with a similar pattern in 2018, and with a decrease to 8 PCUs (61%) in 2019. For LPNs, a high workload pressure was found at 2 PCUs (15%) in 2017, however it increased to 13 PCUs (100%) in 2018, and remained high at 11 of them (85%) in 2019 (Table 4).

**Table 4:**
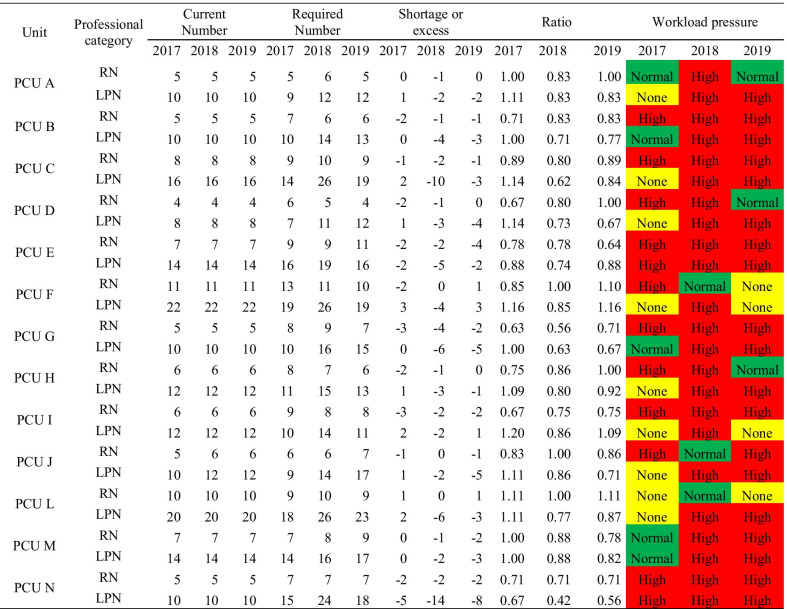
Total required staff based on WISN

RN, registered nurses; LPN, licensed practical nurses

**Fig. 1:**
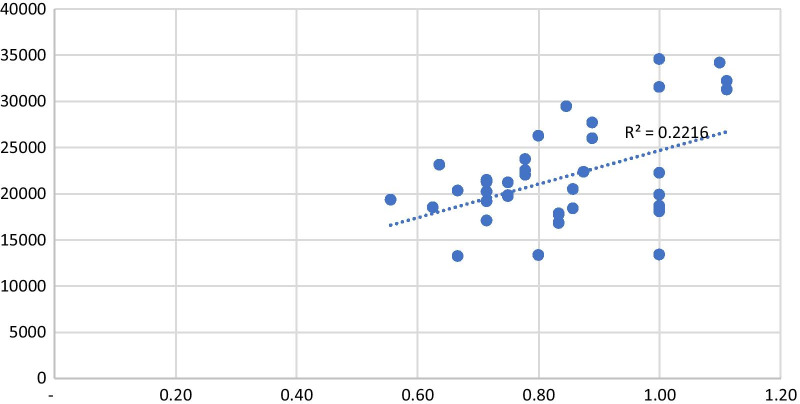
Comparing the registered nurse ratio and number of people registered in each PCU in 2017, 2018 and 2019

**Fig. 2:**
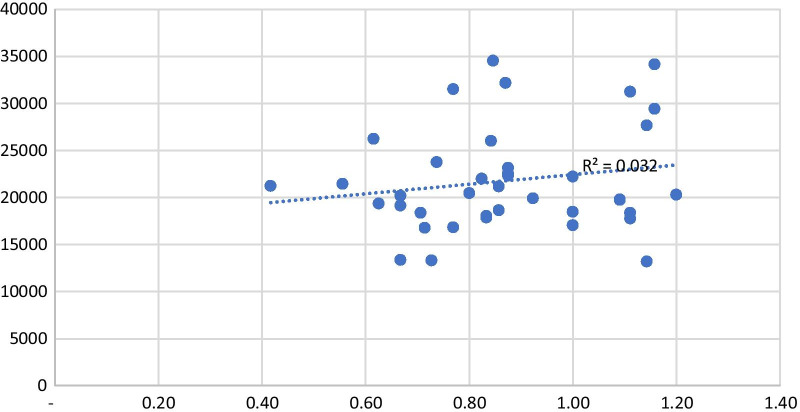
Comparing the licensed practical nurse ratio and number of people registered in each PCU in 2017, 2018 and 2019

There was no statistically significant correlation between workload pressure for RNs and LPNs and the number of people registered per PCU (Figs. 1 and  2).

## Discussion

2020, the International Year of the Nurse and the Midwife, elevated the worldwide recognition of the work performed by nurses and their demands for better working conditions, education, and professional development [12]. In line with this recognition is the fact that nursing plays a critical role in the successful implementation of PHC, which is recognized as the basis of an effective and responsive health system—key to universal coverage [13]. However, the results of this study show how RNs and LPNs working in PHC have been suffering from increasing workload pressure, and the need for better planning and implementation of staffing policies for PHC.

The study identified PCUs with high work pressure and a shortage of nursing professionals needed to meet the actual demand seen during the 3 years of analysis. This is a challenge that PHC must resolve in order to guarantee access and to be able to meet the population’s health needs [14]. The shortage of professionals and its impact should be reported and discussed with PHC teams, so that they can manage high work pressure in the best way possible, balancing access and to promote continuity of care [15]. This scenario of imbalance is known to impact access, generating longer waiting times, which in turn could result in higher mortality rates and other adverse outcomes [16].

In this study, we found no significant association between the number of registered patients and workload pressure. This could be explained not only by the size of PHC teams, but also by additional aspects, such as how frequently patients demand care from PCUs and their teams, which impacts the annual workload and consequently the required number of professionals based on the WISN method. Perhaps more elements, like detailed patient characteristics, are necessary to understand the demand for PHC.

The size of patient panels and the number of PHC teams per service also play an important role, and have been discussed in the literature, including its association with user satisfaction [17]. Ideally, patient panel size should be adjusted to balance the number of professionals on a team and the expected workload. Family physicians have been questioned about the number of patients that could be under their care [15] and the “ideal” patient panel size per physician [18] and advanced methods like machine-learning algorithms have even been tested to answer this question [19]. However, there is little evidence about the “ideal” patient panel size for nursing professionals working as part of a PHC team, such as those established in Brazil and there are investigation gaps about how nursing staff imbalance impact the quality of care in PHC scenario.

Additionally, the distribution of activities among the different professionals that compose the family health team, in spite of presenting common competencies described in the PNAB [6], shows that the actions with the largest impact on the nursing team’s workload, such as vaccination and consultations/appointments, are exclusive to the nurses. However, indirect care activities, such as documentation and administrative meetings related to organizing the work process, are common among team members [20], and can be distributed in such a way that the nurse can dedicate the larger part of their workload to caring for patients.

A study conducted in 2011 [21] that applied the WISN method at a single PCU in São Paulo, Brazil, found that the workload and the number of professionals available was balanced, with a ratio of 0.8 for RNs and 1.0 for LPNs. This study found very different results, and is probably more representative of the real situation, since it included 13 PCUs evaluated for a period of 3 years.

Another study that evaluated workloads of nursing professionals working in PHC teams in Brazil found an association between excessive demand, problems in the physical structure of the units, and gaps in the healthcare network to increased workloads among these professionals, which affected the quality of care and impacted the effectiveness of PHC [22].

The required number of professionals is influenced by a number of variables, including available working time, absences, leave, an adequate information system, the epidemiological background, and the structure of organizational processes at PCUs. These factors were recently put to the test in Brazil during the outbreaks of measles and yellow fever in 2018 and 2019. Nursing professionals working on PHC teams had to organize and implement vaccination strategies in an expedited manner in addition to their routine work [23, 24].

The study presents an increase in work pressure among LPNs between 2017 and 2019, especially in 2019, likely because LPNs started to allocate more of their time to organizing vaccination campaigns and performing household visits to vaccinate, activities that are not accounted for in their productivity evaluation. Moreover, the vaccination calendar has been expanded, and currently includes 19 vaccines for more than 20 diseases [25], with the introduction of new vaccines against the SARS-CoV-2 expected to put even more pressure on PCUs to deliver them in a timely fashion.

As described previously, in Brazil LPNs play a major part in the national immunization program and in preventive actions [26]. The number of nursing professionals can impact the health system’s response to public emergencies, such as vaccination campaigns for both scheduled and pandemic situations, like with COVID-19. This reinforces the importance of applying the WISN method to the planning and implementation of public policies.

Thus, to understand the current deficit of LPNs it would be relevant to plan staffing needs in the face of a probable high demand for vaccination campaigns, and for future analysis that could take into account seasonal variations and target coverage rates that can be used to plan, improve allocation, and when necessary, increase the number of professionals during strategic periods. Historical patterns that characterize an increase in demand or a reduction in AWT can be analyzed to determine strategic periods. This is why it is important for the WISN method to be applied dynamically and relevantly with periods of rapid and assertive actions throughout the year.

As in other parts of the world, an aging population and the shift of the disease burden from infectious to noncommunicable diseases are important factors impacting the demand for PHC services and the volume of RNs’ scheduled and unscheduled appointments in Brazil [27]. An observational study conducted in Brazil found that RNs working as part of a PHC team spent 11.6% of their working time with appointments [11], which matches the high volume found for the PCUs included in this study.

In addition, the reduction in activities over the years, probably associated with time spent on unscheduled appointments, is a notable finding. This could be explained by the implementation of advanced access scheduling at the PCUs included in the study, which began [14] in 2017, with RNs’ unscheduled appointments accounting for up to 70% of their agenda.

The evaluation of staffing needs could also work as an ongoing education and quality improvement strategy, since annual analyses provide an opportunity to reflect on the practice and organization of nursing professionals. A study that evaluated the implementation of the WISN method to two PHC teams in the state of São Paulo found that it promoted a change in the team’s attitude towards the correct recording of data in information systems, and prompted a reorganization of the territory covered by each team [28].

Thus, staff planning with the WISN method allows for an important reflection, considering that not all of the problems at the PCUs are related to the amount of professionals, just as the amount of professionals is not a solution to all of the problems. Analyzing the data that make up the calculation makes it possible to recognize the obstacles in organizing work, distributing activities among team members, the amount of activities performed annually, the amount of absences for health leave and/or other types of leave that historically contribute to understanding the pressure on the team’s workload due to dynamic patient demand.

In this way, staff planning at PHCs is a strategic process, balancing demand and supply through the analysis of the availability staff and their work process, and through a systematic planning method that aims for continual improvement in the process of ongoing education and implementation of public policy.

Studies using the WISN contribute prominently to the discussion on staff planning at PHCs as a tool for public policy that facilitates management decisions and thus allows for the strengthening of universal health coverage, considering the key role that professionals like RNs and LPNs play in the results and increase of health access.

One of the main limitations of this study is the fact that it only included data recorded for patients that accessed the PCUs during the study period. PHC in Brazil is organized geographically, with the assignment of catchment areas to PCUs and PHC teams, with no overlap or gaps between them. Although PCUs are responsible for the entire population living in their catchment area, some proportion of these individuals never or rarely access services, and are not represented in productivity-based analyses, such as those conducted in this study. Therefore, the study results reflect nursing staffing needs based on the population that accessed services, and not on the entire covered population, an additional challenge that needs to be addressed in future studies.

## Conclusion

This study found that RNs and LPNs, working as part of PHC teams in Brazil, experienced high workload pressures, which could be associated with the epidemiological background, the structure of organizational processes, and the flexibility of policies determining the composition and number of professionals on PHC teams.

The PCUs should work as the first point of contact with the health system, while ensuring equitable access and quality of care. The appropriate staffing of PHC teams represents a key activity in order to be successful in that mission, and the WISN method could be a useful tool to support this process.

## Data Availability

The data that support the findings of this study are available from the corresponding author upon reasonable request.

## Abbreviations

PHC: Primary health care
WISN: Workload Indicators of Staffing Need
FHS: Family Health Strategy
PNAB: National Policy for Primary Care (PNAB, acronym in Portuguese)
RN: Registered nurse
LPNs: Licensed practical nurses
AWT: Available working time
PCU: Primary Care Unit
